# Open and closed loops: A computational approach to attention and consciousness

**DOI:** 10.2478/v10053-008-0096-y

**Published:** 2012-02-03

**Authors:** Sabrina Trapp, Henning Schroll, Fred H. Hamker

**Affiliations:** 1 Department of Neurology, Max Planck Institute for Human Cognitive and Brain Sciences, Leipzig, Germany; 2 Charite University Hospital, Bernstein Center for Computational Neuroscience, Berlin, Germany; 3 Department of Computer Science, Chemnitz University of Technology, Chemnitz, Germany

**Keywords:** attention, consciousness, interdependence, computational model, closed loops, open loops

## Abstract

Within recent years, researchers have proposed the independence of attention and consciousness on both empirical and conceptual grounds. However, the elusive nature of these constructs complicates progress in the investigation of their interaction. We present a framework within which we conceptualize attention and consciousness in computational terms. Here, the concepts are consi-dered as large-scale, functionally and structurally different processes, embedded in a biologically inspired architecture, spanning the full arc from stimulus to response. Our architecture assumes a general independence of attention and consciousness, but supposes strong interactions. Furthermore, it addresses the developmental aspect, stressing that these functions have to gradually develop through learning.

## INTRODUCTION

Imagine you are watching Shakespeare’s *Hamlet* in a theater. Afterwards, a friend asks you whether you noticed that the skull was from a sheep. You might reply: “No, I didn’t pay attention to the skull”. Alternatively, you could state that you didn’t consciously perceive this detail. Based on phenomenological experience in our daily lives, we rarely distinguish between being in an *attentive* and being in a *conscious* state of mind. Intuitive meshing of these constructs has also influenced scientific conceptualization. For instance, it has been assumed that attention acts as a mechanistic precursor of conscious experience (Mack & Rock, 1998; Posner, 1994). Multiple empirical studies support the idea that our ability to detect and become consciously aware of visual changes strongly depends on the involvement of attention (O’Regan, Deubel, Clark, & Rensink, 2000; Rensink, O’Regan, & Clark, 1997, 2000; Simons & Chabris, 1999). Additionally, some neural structures and their mutual connections subserving attention and consciousness appear to closely overlap (Rees & Lavie, 2001).

Recently, the relationship between attention and consciousness has sparked new interest. Based upon findings that subjects are able to categorize scenes in the near absence of top-down attention (Li, VanRullen, Koch, & Perona, 2002; Reddy, Reddy, & Koch, 2006; Reddy, Wilken, & Koch, 2004), it was proposed that the constructs are less interwoven than had previously been assumed (Koch & Tsuchiya, 2007; van Boxtel, Tsuchiya, & Koch, 2010). However, conceptual problems impede an unequivocal decision as to whether attention and consciousness are independent or meshed. Neither attention nor consciousness are well-defined, unitary constructs. The construct of *attention*, for instance, can be divided into feature-based, object-based, and spatial attention (i.e., by the kind of selection), into bottom-up and top-down attention or into focused and diffuse attention. Koch and Tsuchiya (2007) therefore limited their discussion about the independence of attention and consciousness to (spatial) visual top-down attention. However, it is still a matter of debate whether and to what extent even visual top-down attention is a unitary construct. Thus, it was suggested that “relatively independent attentional mechanisms operate within different cognitive subsystems depending on the demands of the current stimuli and tasks.” (Woodman, Vogel, & Luck, 2001, p. 153). Attention then rather constitutes an umbrella term, or a catch-all-term (Chun, Golomb, & Turk-Browne, 2011) that characterizes control processes in perception and cognition operating at multiple stages of the system (Kastner & Pinsk, 2004).

Similarly, the concept of *consciousness* is far from being well defined. There are at least two components of consciousness that are frequently debated (Block, 1995): While *phenomenal consciousness* is defined as the content of an experience, *access consciousness* refers to the process whereby information is made available to the brain’s “consumer” systems (e.g., planning or evaluation systems; Block, 2005). Although the latter is a functional term and therefore in principle amenable to emulation in synthetic systems, it remains a rather elusive construct. For example, it was further subdivided into *awareness-access* and *broadcasting-access* (Block, 2007).

In order to avoid the risk of ending in a merely “semantic debate” without much practical gain, precise conceptualizations are needed. As computational models require an exact definition of each of their underlying processes, it might be of use to approach *attention* and *consciousness* from a computational perspective. We will here argue that - from such a computational perspective - attention and consciousness might be understood as open- and closed-loop processes, respectively. Although parts of our framework are already implemented (Schroll, Vitay, & Hamker, 2012; Vitay & Hamker, 2010), the term *framework* is thought to do justice to the fact that it still contains gaps to be filled by future research.

## A Computational Account of Attention and Consciousness

From a functional point of view, the purpose of attention is to avoid informational overload of the brain’s limited processing capacity (Broadbent, 1971) or alternatively, to support parameter specification for action (Allport, 1987; Neumann, 1990). This is thought to be achieved through filtering of information which logically implies some form of selection. There is evidence from neurophysiology that selection is implemented through a competitive biasing process, in which processing resources are allocated to relevant stimuli (Desimone & Duncan, 1995). Relevance is either defined by the task (task-driven or top-down attention), or by the saliency of a stimulus (stimulus-driven or bottom-up attention). We showed elsewhere that attentional effects in exogenous cuing and motion onset experiments (e.g., Posner & Cohen, 1984; Yantis & Hillstrom, 1994) can be explained by simple sensory feedback loops (Zirnsak, Beuth, & Hamker, 2011). Accordingly, in our view, the major computational purpose of attention is to determine the top-down bias through which relevant information is selected.

It was suggested that the computational goal of visual consciousness is to provide the best interpretation of the current scene (Crick & Koch, 1995, 1998). In this sense, the focus is on information that is already present and on its further cycling within the system to accomplish a task at hand. There is gathering consensus that conscious information processing engenders global availability of information and wide-spread, distributed processing in the brain (Dennett, 2001; Kanwisher, 2001; Varela, Lachaux, Rodriguez, & Martinerie, 2001). This idea was first outlined within the framework of global workspace theory (Baars, 1988) and was recently extended to the neuronal level of analysis (Dehaene & Naccache, 2001).

In summary, the networks involved in attention serve to select relevant information, while the networks subserving conscious processing determine which elements of information should be a part of the “global workspace”. In the following, we will build upon these ideas and argue that it might be useful to think of *attention* as a mechanism responsible for providing a selective bias within open cortico-subcortical loops while consciousness might be understood as a process of activating memory and stimulus content within closed cortico-subcortical loops.

### General framework

In our computational framework, we postulate the existence of two kinds of cortico-subcortical loops. Closed loops connect a specific ensemble of cortical cells via subcortical structures back to itself. Open loops connect a cortical ensemble to a different one (which may or may not be in the same cortical region). In general, both closed and open loops involve the thalamus which is heavily connected with the cortex and is of vital importance for both driving and modulating cortical processing (Sherman & Guillery, 2005). A basic kind of open or closed loop therefore consists of mutually excitatory connections between cortex and thalamus.

We propose that the *development* of connectivity in such loops involves additional subcortical structures, particularly the basal ganglia (BG), but probably also hippocampus and cerebellum. The BG are part of parallel cortico-BG-thalamo-cortical loops that contribute to different functional domains (Alexander, DeLong, & Strick, 1986). These loops interact hierarchically to allow information flow from motivational loops via motor-planning loops to motor-execution loops of the brain (Haber, 2003). We suggest that BG are particularly important for learning functional connectivity within such loops but might also remain in charge for online control: Based on reinforcement signals, they will learn to integrate inputs from distinct cortical areas (e.g., from cortical areas related to sensory, cognitive, motor, and motivational functions) to induce activity within specific open or closed loops. Once BG have learned such an integration for a particular combination of inputs, they continue to perform it until cortico-thalamo-cortical or cortico-cortical connections may take over control (Ashby, Ennis, & Spiering, 2007; Waldschmidt & Ashby, 2011).

#### Closed Loops

**Figure 1.**
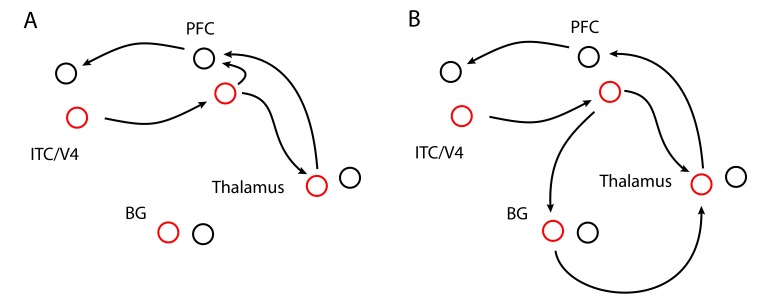
Closed loops of different complexities, here for visual processing. Cell ensembles are shown as circles. Colors indicate which (of two) representations a cell is coding. Closed loops allow representation-specific activity to reverberate within self-excitatory circles. Section A: Closed cortico-cortical and cortico-thalamo-cortical loops. Section B: Closed cortico-cortical and cortico-basal ganglio-thalamo-cortical loops. BG = basal ganglia. ITC = inferior temporal cortex. PFC = prefrontal cortex. V4 = visual area V4.

Closed loops (see Figure 1) allow information to reverberate within them. We hypothesize that consciousness arises from the activation of relevant limbic, associative, or motor loops which ensure the integration and maintenance of information in a global workspace.

Recently, we developed a computational model that learns to maintain information in cortico-BG-thalamo-cortical loops (Schroll et al., 2012). In this model, the BG are crucial for *activating* closed loops and, most importantly, for *learning* task-relevant associations (Figure 1, Section B). Without learning these associations, habitual behavior becomes a difficult, potentially impossible task. The transition from deliberate behavioral control to habit formation could thus provide important insights into the functional role of consciousness, particularly since habitual behavior appears to correlate both with the withdrawal of BG from processing (Miller & Antzoulatos, 2011; Pasupathy & Miller, 2005; Waldschmidt & Ashby, 2011) and with a decrease of conscious control. We do not propose that consciousness “resides” in the BG, but that they are fundamentally involved in making contents globally *available* by their control over thalamo-cortical loops. This control could be either indirect, through learning (i.e., by progressively interlinking loops), or it could be direct, through online control (i.e., when the BG stay in charge for switching thalamo-cortical loops on and off). Consistent with such a prominent role of BG in consciousness, a recent fMRI study showed decreasing levels of consciousness (induced by propofol) to be accompanied by a decrease in functional connectivity between the putamen and other brain regions, while relatively preserving thalamo-cortical connectivity (Mhuircheartaigh et al., 2010). The authors conclude that a disruption of subcortical thalamo-regulatory systems (involving the BG) may precipitate a disruption of thalamo-cortical connectivity.

Generally, when stimuli or events are processed, different contents will be processed within different loops. The perception of color and shape of an object, for instance, the feelings when touching it and the memories and associations linked to it, will likely be analyzed within separate loops. To encode the contents of visual consciousness, closed loops involve modality-specific areas, such as visual area V4 and inferior temporal cortex (ITC; analogously, for audition, the superior temporal gyrus would be involved). Although mid-level visual areas project into the BG as well (Seger & Miller, 2010), closed-loop processing (cf. Figure 1) particularly involves prefrontal cortex (PFC). As illustrated by Schroll et al. (2012), prefrontal neurons can provide context information to closed cortico-BG-thalamo-cortical loops. Thus, PFC is an important interface between motivation, sensory representations, and action by providing task-relevant constraints for decision processes.

If consciousness depends upon closed-loop activity, how can unconscious processing be described in our framework? Several studies demonstrated that subliminally presented stimuli are still processed in the visual system, even with regard to semantic meaning (Dehaene et al., 1998; Kiefer, 2002; Kiefer & Spitzer, 2000). In accord with this, our framework does not require closed-loop recurrent activity anywhere in the system for a spread of activation from lower-order to higher-order brain regions (e.g., via open loops). In this early spread of activation, masked and unmasked stimuli are not yet distinguishable by their neural response (Kovács, Vogels, & Orban, 1995; Rolls, Tove, & Panzeri, 1999; Thompson & Schall, 2000). We propose that unconscious processing is likely to derive from cessation of stimulation before stimulus-related activity has passed through relevant closed loops. In this situation, the neural trace decays before the activity begins to cycle. The minimum presentation time for a stimulus to be consciously perceived can therefore be defined as the time needed by a piece of information to travel through the corresponding closed loops once. This could occur in events of 100 ms, as suggested by Wu, Busch, Fabre-Thorpe, and Van Rullen (2009).

#### Open Loops

**Figure 2.**
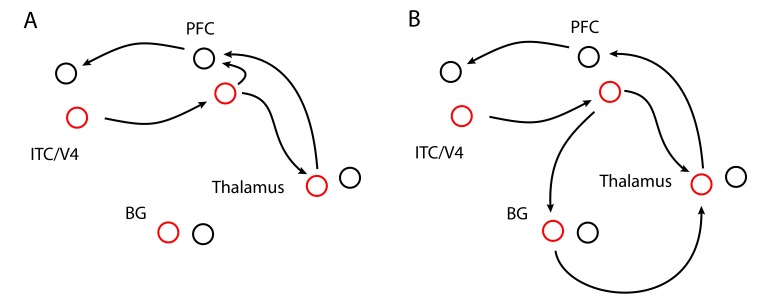
Open loops of different complexities. Cell ensembles are shown as circles. Colors indicate which (of two) representations an ensemble is coding. Open loops allow activity to spread from one set of representations to a different one. Section A: Open cortico-cortical and cortico-thalamo-cortical loops. Section B: Open cortico-basal ganglio-thalamo-cortical loops. BG = basal ganglia. ITC = inferior temporal cortex. PFC = prefrontal cortex. V4 = visual area V4.

Open loops (cf. Figure 2) allow selective biasing of information processing in a unidirectional way. Desimone and Duncan (1995) stress the importance of top-down biasing signals for attentional selection in their neurophysiologically motivated framework of attention. However, “biased competition” is a single-neuron theory and hence offers no specification of how this process is implemented in a full network, for example by specifying the sources of such biasing signals. The prefrontal cortex is known for its involvement in executive functions and is a good candidate for such biasing signals because of its anatomical connectivity (Miller & Cohen, 2001). Recently, we provided a computational framework to explain how attentional processes can derive from open-loop functioning (Vitay & Hamker, 2010). In this model, prefrontal, task-related representations (via cortico-BG-thalamo-cortical loops) bias a competitive interaction between different stimulus representations within the inferior temporal cortex (ITC) in a top-down manner. Depending on which PFC representations are active at a given time, different ITC representations will receive selective excitation. The PFC therefore modulates ITC processing by selectively favoring specific representations. The idea of open-loop modulation can easily be generalized to take place between any two cortical ensembles, not only for the visual, but also for the tactile or auditory domain. In computational terms, we thus conceptualize *attention* as a process where a specific ensemble of cells activates another ensemble via an open loop in a task-related manner.

### Interactions between open and closed loops

In our framework, *consciousness* and *attention* are disjunct in that they rely on different computational processes. However, this does not exclude the possibility that they interact with and depend upon each other. The activation of closed loops is dependent upon present sensory input as forwarded via open loops. Thus, open-loop activation usually *precedes* closed-loop activation in the sense that it boosts a stimulus representation that is supposed to cycle in a closed loop. Similarly, Dehaene and Nacchache (2001) proposed that attention acts as an amplifier to allow selected information to become part of the global workspace. In our model, open-loop biasing can occur independently of closed-loop processing. This is in line with several experiments which demonstrate that attentional sets can modulate *unconscious* semantic and visuo-motor processing pathways (Kiefer & Brendel, 2006; Kiefer & Martens, 2010; Martens, Ansorge, & Kiefer, 2011) and do not necessarily lead to conscious experiences (Kentridge, Nijboer, & Heywood, 2008; Naccache, Blandin, & Dehaene, 2002; Tapia, Breitmeyer, & Shooner, 2010). Furthermore, interactions can also occur in the opposite direction. We demonstrated elsewhere that closed cortico-BG-thalamo-cortical loops can learn to maintain information, and that this information, in turn, can be used to bias action selection via open loops (Schroll et al., 2012).

### Learning of consciousness and attention

From our perspective, attention and consciousness are not fully innate. Rather, they evolve as an organism interacts with its environment. Although novel stimuli may be perceived consciously upon first encounter and may also drive attention in a bottom-up way, we assume these processes to depend heavily upon prior experiences with similar stimuli. We do not argue against the possibility that some basic mechanisms of consciousness and (stimulus-driven) attention may depend upon pre-wiring, but we want to emphasize that early learning has an important and often underestimated impact.

According to our computational framework, reinforcement-driven structural changes in BG guide the development of thalamo-cortical connections as required to receive positive reinforcements (Schroll et al., 2012). Similarly, Vitay and Hamker (2010) demonstrated that open cortico-BG-thalamo-cortical loops can learn to extensively bias processing within sensory cortex to subserve attentional selection. There is indeed mounting evidence of the involvement of BG in learning cognitive tasks (Packard & Knowlton, 2002). Even more, there is evidence from monkey experiments that associative learning in the striatum precedes learning in the prefrontal cortex (Miller & Antzoulatos, 2011; Pasupathy & Miller, 2005). Thus, we suggest that the development of cortico-BG-thalamo-cortical loops is a necessary prerequisite for conscious and attentional processing.

## Discussion

We here outlined a preliminary computational framework within which attention and consciousness can be understood as open- and closed-loop processes, respectively. Our proposal is largely in agreement with previous theories of attention and consciousness, but provides a computational and therefore more precise taxonomic framework of attention and consciousness. It particularly emphasizes the role of BG and the importance of learning. In our theoretical framework, attention and consciousness are independent but strongly interacting processes. In the following, we briefly outline how the concepts of open and closed loops relate to the key tenets of previous models of attention and consciousness.

### Open loops and theories of goal-directed attention

It is generally assumed that goal-directed attention requires top-down control for biased competition (Desimone & Duncan, 1995; Miller & Cohen, 2001). While several computational models of attention rely on such principles, we previously proposed a systems-level model of attention that fleshes out the rather abstract concept of biased competition by demonstrating how competitive effects emerge by interacting brain areas (Hamker, 2005a, 2005b; Hamker & Zirnsak, 2006; Zirnsak et al., 2011). In this model, top-down signals influence the neural dynamics of a network consisting of areas V4 and ITC as well as prefrontal areas including the frontal eye field. We further showed that, dependent on a cue, BG can learn to perform delayed match-to-sample and delayed pair-association tasks (Vitay & Hamker, 2010). In these tasks, an object is presented, followed by the cue. Then, a choice display containing two objects is presented. Dependent on the cue, reward is given if either the same object or a pair-object is chosen. Through dopamine-modulated learning, the prefrontal cortex learns to provide top-down signals to bias processing in a sensory cortico-BG-thalamo-cortical loop. Generalizing from these computational studies, we refer to attention as the delivery of appropriate top-down signals for visual guidance via open loops, regardless of whether they relate to basic or higher-level contents.

### Closed loops and theories of consciousness

In our framework, *consciousness* is conceptualized as closed-loop processing which is similar to ideas of reentrant processing that is considered crucial in most theoretical accounts of consciousness (Crick & Koch, 2003; Dehaene, Sergent, & Changeux, 2003; Di Lollo, Enns, & Rensink, 2000; Edelman, 1989; Edelman, Gally, & Baars, 2011; Grossberg, 1999). Lamme and Roelfsema (2000) suggested to use the feedforward-feedback dichotomy to conceptualize the difference between unconscious and conscious vision. A central tenet of this proposal is that recurrent processing within the visual cortex is thought to be sufficient for (phenomenal) consciousness (Lamme, 2006). Although this idea is generally consistent with our framework, we do not consider all forms of reentrant processing essential for consciousness. For example, our computational model of attention (Hamker, 2005a, 2005b) heavily relies on reentrant processing (which serves to boost relevant information), but does not explain consciousness. Similarly, we do not fully agree with the suggestion to regard information integration as the quintessence of consciousness (Tononi, 2004). We showed that information integration can already take place in pre-conscious processing (Hamker, 2005a, 2005b) and that (attentional) reentrant processing (e.g., from ITC to V4 and from frontal eye fields to V4) enforces binding and ensures that different brain areas process different aspects of the same content (or physical object in the world). In our view, without the specification of an underlying neuroanatomical and functional architecture, both recurrent processing and information integration remain necessary, but by no means sufficient correlates of consciousness. This position is motivated by the fact that a single neuronal structure or computational process can usually be related to more than one psychological concept or domain: Persistent activity, for instance, is traditionally associated with working memory but may also be linked to decision making (Curtis & Lee, 2010). Moreover, individual brain structures can be associated with a broad range of processes (e.g., Duncan & Owen, 2000; Fadiga, Craighero, & D’Ausilio, 2009). An example is the discovery of a close overlap between the neural systems underlying attention and working memory (LaBar, Gitelman, Parrish, & Mesulam, 1999; Mayer et al., 2007). This could be considered as a “specifity problem” in cognitive neuroscience. In this sense, it will be very difficult to identify neuronal correlates that unequivocally refer to consciousness, that is, those that will not be part of related constructs such as working memory or decision making. Therefore, instead of identifying singular neural correlates of consciousness, we specify the computational principles of an extensive anatomical architecture, providing both necessary and sufficient conditions for consciousness.

Dehaene and Naccache (2001), in their neural model of global workspace theory, also offer an extensive neural architecture. In this model, a global workspace is implemented by reentrant processing within specialized modules. In contrast, we emphasize the role of the BG and the aspect of learning. Finally, we not only combine attentional and conscious processing in one framework, but also offer more tangible taxonomies for these phenomena.

Most importantly, our architecture is driven by a top-down engineering approach, that is, we incrementally build an artificial system that simulates the properties associated with consciousness and related constructs. This circumvents some methodological shortcomings associated with experimental procedures such as functional magnetic resonance imaging (fMRI). The problem is that “the statistical analyses and thresholding methods applied to the haemodynamic responses probably underestimate a great deal of actual neural activity related to the stimulus or task” (Logothetis, Pauls, Augath, Trinath, & Oeltermann, 2001, p. 154). In other words, it can never be ruled out that additional structures are involved in the neural correlates of consciousness (NCC). In contrast, our computational model allows to observe how certain dynamics *evolve* and which structures are necessary for a task at hand. Hence, it makes clear predictions that can, in turn, guide a more theory-driven design and analysis of fMRI experiments.

We here primarily addressed the computational correlates of consciousness. This is often referred to as the “easy problem”, while the “hard problem” denominates the difficulty of explaining why we have qualitative phenomenal experiences (Chalmers, 1995). The latter is sometimes thought to be by its very nature not a tangible subject for scientific investigations (Nagel, 1974). However, we do not imply that our framework excludes phenomenological experiences or qualia (e.g., the feeling of seeing a color such as red). We rather assume that this will require additional closed-loop processing by limbic thalamo-cortical loops in which the BG also participate (Humphries & Prescott, 2010; Vitay & Hamker, 2011).

## Conclusion

One major problem in consciousness research so far has been that *consciousness* and *attention* are not well defined. By suggesting open and closed loops to differentiate these constructs, we here provide more precise computational conceptualizations. We developed a patchwork of computational models (Hamker, 2005a, 2005b; Schroll et al., 2012; Vitay & Hamker, 2010) which, taken together, not only provide a framework of attention and consciousness in terms of cortico-BG-thalamo-cortical open and closed loops, but also specify how these processes interact. In agreement with existing theories of attention and consciousness, we outline how properties such as biased competition, recurrent activity, and information integration arise as a consequence of network dynamics. Our framework could provide guidance to experimental researchers and allows computational simulations of experiments.

Most importantly, our framework of open and closed loops not only specifies computational mechanisms but also embeds them in a detailed neuroanatomical architecture and thus circumvents the specificity problem in cognitive neuroscience (e.g., Curtis & Lee, 2010; Duncan & Owen, 2000). Finally, our approach stresses the often overlooked developmental aspect of attention and consciousness as it offers an interpretation of how attentional biasing and conscious processing emerge to optimally adapt an agent to its environment. We admit that our framework is far from being complete, but hope, in the style of Warren McCulloch, that the reader will not bite our fingers, but look where we are pointing.
